# Cumulative burden of inflammation predicts colorectal neoplasia risk in ulcerative colitis: a large single-centre study

**DOI:** 10.1136/gutjnl-2017-314190

**Published:** 2017-11-17

**Authors:** Chang-Ho Ryan Choi, Ibrahim Al Bakir, Nik-Sheng (John) Ding, Gui-Han Lee, Alan Askari, Janindra Warusavitarne, Morgan Moorghen, Adam Humphries, Ana Ignjatovic-Wilson, Siwan Thomas-Gibson, Brian P Saunders, Matthew D Rutter, Trevor A Graham, Ailsa L Hart

**Affiliations:** 1 St Mark’s Hospital, London, UK; 2 Centre for Tumour Biology, Barts Cancer Institute, Queen Mary University of London, London, UK; 3 Gastroenterology, University Hospital of North Tees, Stockton-on-Tees, UK

**Keywords:** surveillance, colorectal cancer screening, inflammation, colorectal neoplasia, colonoscopy

## Abstract

**Objective:**

Ulcerative colitis (UC) is a dynamic disease with its severity continuously changing over time. We hypothesised that the risk of colorectal neoplasia (CRN) in UC closely follows an actuarial accumulative inflammatory burden, which is inadequately represented by current risk stratification strategies.

**Design:**

This was a retrospective single-centre study. Patients with extensive UC who were under colonoscopic surveillance between 2003 and 2012 were studied. Each surveillance episode was scored for a severity of microscopic inflammation (0=no activity; 1=mild; 2=moderate; 3=severe activity). The cumulative inflammatory burden (CIB) was defined as sum of: average score between each pair of surveillance episodes multiplied by the surveillance interval in years. Potential predictors were correlated with CRN outcome using time-dependent Cox regression.

**Results:**

A total of 987 patients were followed for a median of 13 years (IQR, 9-18), 97 (9.8%) of whom developed CRN. Multivariate analysis showed that the CIB was significantly associated with CRN development (HR, 2.1 per 10-unit increase in CIB (equivalent of 10, 5 or 3.3 years of continuous mild, moderate or severe active microscopic inflammation); 95% CI 1.4 to 3.0; P<0.001). Reflecting this, while inflammation severity based on the most recent colonoscopy alone was not significant (HR, 0.9 per-1-unit increase in severity; 95% CI 0.7 to 1.2; P=0.5), a mean severity score calculated from all colonoscopies performed in preceding 5 years was significantly associated with CRN risk (HR, 2.2 per-1-unit increase; 95% CI 1.6 to 3.1; P<0.001).

**Conclusion:**

The risk of CRN in UC is significantly associated with accumulative inflammatory burden. An accurate CRN risk stratification should involve assessment of multiple surveillance episodes to take this into account.

**Significance of this study**

**What is already known about this subject?**

- While severity of inflammation is recognised as an important risk factor for developing colorectal neoplasia (CRN) in ulcerative colitis (UC), an impact of overall inflammatory burden accumulated over the duration of colitis is poorly understood.
- This is an important factor to consider, because the current British Society of Gastroenterology (BSG) guideline suggests assessing the severity of inflammation using the most recent colonoscopy alone in determining the next surveillance interval (i.e. without requiring assessment of previous colonoscopies). This approach may not accurately represent an individual’s accumulated inflammatory burden.

**What are the new findings?**

- Accumulative inflammatory burden was strongly associated with CRN risk, with 2-fold increase in risk for approximately 10, 5 or 3.3 years of continuously mild, moderate or severe active microscopic inflammation, respectively.
- Reflecting this, inflammation-based risk stratification was only sufficiently predictive when information from multiple colonoscopies was used—severity of inflammation in the most recent colonoscopy alone was not significantly associated with CRN risk.
- The mean severity of microscopic inflammation derived from all surveillance procedures performed in the last 5 or 10 years (consisting of approximately 3–4 colonoscopies) is an accurate marker of the CRN risk and can be rapidly calculated in a busy clinical practice.
- Patients with persistent inflammation have a significant risk of developing CRN, regardless of it’s severity

**How might it impact on clinical practice in the foreseeable future?**

- The CRN risk stratification should involve assessment of multiple surveillance episodes to take into account the effect of cumulative inflammatory burden: the severity of microscopic inflammation averaged over the preceding 5 or 10 years may offer an accurate and pragmatic risk stratification strategy.
- Patients with severe or persistent disease should be offered more frequent surveillance and actively treated to achieve mucosal healing in order to reduce future risk of CRN.

## Introduction

Patients with ulcerative colitis (UC) have a significant risk of developing colorectal cancer (CRC).^1–3^ Decades of research have revealed that inflammation has a central role in carcinogenesis, as evidenced by a close relationship between the CRC risk in UC and inflammation-related risk factors such as duration of colitis and^1 2^ severity of inflammation.^4–6^ In light of these findings, the international guidelines have recommended increasing the frequency of colonoscopic surveillance on the basis of increasing disease duration^7^ or worsening degree of active inflammation seen in the most recent colonoscopy.^8^

UC is a life-long illness characterised by repeated episodes of relapse and remission. As a consequence, the severity of disease activity may vary significantly over time and accumulated inflammatory damage may differ considerably among individual patients. This important fact is not fully accounted for by current risk stratification used in our clinical practice. For example, the duration of colitis—a proxy measure for cumulative inflammatory insult over time—assumes that the degree of inflammatory activity stays constant over time. This is clearly not the case as accumulated inflammatory damage is unlikely to be the same between patients who suffer persistently active disease and those who have predominantly quiescent disease. Similarly, assessing the severity of inflammation in the most recent colonoscopy, the strategy endorsed by the British Society of Gastroenterology guideline (BSG)^8^ provides only a snapshot of an individual’s disease history and may overlook significant inflammatory insults occurring previously that could have an important impact on carcinogenesis.

To understand the validity of these risk factors, it is crucial to ascertain the impact of summative inflammatory damage in the colonic mucosa accumulated over time during a patient’s course of disease on carcinogenesis. In this study, we examined the association between the cumulative inflammatory burden (CIB) and the risk of developing colorectal neoplasia (CRN) in patients with UC and assess how it compares to that of known risk factors, namely duration of colitis and severity of inflammation based on a single recent colonoscopy. Building from this, we suggest a pragmatic inflammation-based risk stratification strategy that could be used to accurately predict the risk of developing CRN in patients with UC.

## Methods

### Surveillance programme

St Marks' hospital is a tertiary referral centre in the UK. Patients with endoscopic and histological evidence of UC proximal to the splenic flexure were offered surveillance colonoscopies every 1–2 years from 8 to 10 years after the onset of UC symptoms. At each colonoscopy, segmental random biopsies were taken, with additional targeted biopsies from any suspicious areas.

Each episode of dysplasia was graded according to the 1983 Inflammatory Bowel Disease Dysplasia Morphology Study group classification^9^ and was independently reviewed by two experienced gastrointestinal pathologists at the time of diagnosis in accordance with the standard hospital policy.

### Patient identification, inclusion and exclusion criteria

Patients were identified from the St Mark’s hospital IBD database. The index colonoscopy was defined as the first colonoscopy performed at our centre subsequent to the patient entering the surveillance programme after 8 years of colitis symptom duration.

Patients were included in the study if they had: (1) histologically confirmed extensive UC (inflammation proximal to the splenic flexure), (2) a surveillance colonoscopy between January 2003 and December 2012 and (3) at least one or more subsequent surveillance procedures. Patients were excluded if they had known dysplasia or CRC prior to or at the time of first surveillance colonoscopy at our centre, disease duration <8 years (unless the patient had a history of primary sclerosing cholangitis (PSC)) or diagnosed to have Crohn’s disease or indeterminate colitis.

For patients who met above inclusion criteria, we considered all available endoscopy episodes.

### Data collection and study end point

Data on potential predictors were collected from the hospital’s IBD database, clinical notes, endoscopy and histology reports. The study endpoint was defined as development of CRN during surveillance or at colectomy up to 1 July 2013. CRN was defined as development of high-risk low-grade dysplasia (LGD), high-grade dysplasia (HGD) or CRC. High-risk LGD was defined as LGD confirmed in one of the following lesions: (1) non-polypoid in shape (ie, Paris type II, III, irregular, diffuse or plaque-like lesions) or (2) large (≥1 cm).^10^

If the patient had not developed CRN, they were censored at the earliest of: the time of the latest available surveillance colonoscopy, colectomy or 1 July 2013.

### Calculation of cumulative inflammatory burden

Each colonoscopy episode was assigned with two single inflammation scores:

- *Endoscopic inflammation score*: derived from severity of macroscopic inflammation reported in the segment with the worst degree of documented inflammation present within any segment of the colon. This was based on endoscopy report at the time of the procedure. Four-point scale shown below was used.

- Normal or quiescent disease (=0).
- Mild active inflammation (=1).
- Moderate active inflammation (=2).
- Severe active inflammation (=3).

- *Histological inflammation score*: derived from severity of microscopic inflammation reported in the segment with the worst degree of documented inflammation present within any segment of the colon, in accordance with the pathologist’s report at the time of histological examination. Similarly to the endoscopic score, a 4-point scale shown below was used.

- Normal or quiescent disease (no inflammatory cells or chronic inflammation only=0).
- Mild active inflammation (cryptitis but no crypt abscesses=1).
- Moderate active inflammation (few crypt abscesses=2).
- Severe active inflammation (numerous crypt abscesses=3).

**Figure 1:**
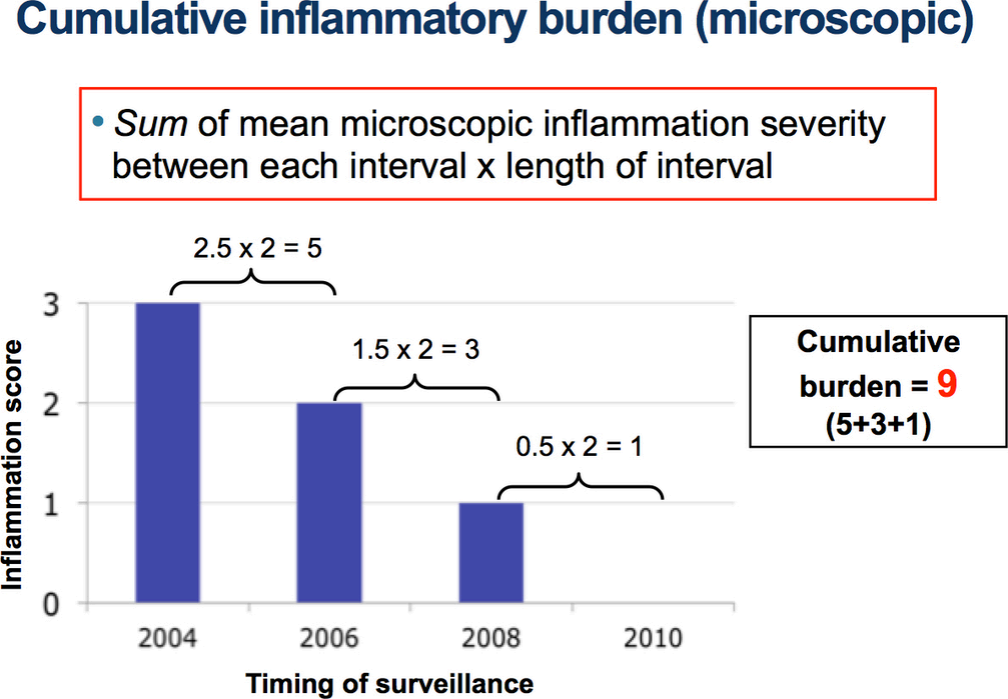
An illustration of how CIB for each patient was calculated. This patient had severe (inflammation score=3), moderate (score=2), mild active (score=1) and then quiescent microscopic disease (score=0) in 2004, 2006, 2008 and 2010, respectively. The CIB for the first surveillance interval (ie, from 2004 to 2006) would be an average microscopic severity between the two surveillance episodes (ie, 3+2/2=2.5) multiplied by the length of surveillance interval (2 years), which is 5. The overall CIB was obtained by summing the CIB scores from all surveillance intervals: so in this patient, the overall CIB is 9. CIB, cumulative inflammatory burden.

Once these scores were derived, CIB was calculated for each surveillance interval by multiplying average severity score between a pair of surveillance episode by the length of surveillance interval in years (an example is shown in figure 1).

### Time-dependent covariates and data analysis

Data analyses were performed using the R statistical software package  (R Development Core Team,  2008).^11^ All continuous variables were reported as medians with interquartile ranges (IQRs). The association between each variable and the study end points was examined using time-dependent Cox proportional hazards methods with right-censored data. The detailed information on statistical methods used to construct multivariate models and how each variable was categorised in a time-dependent Cox-model is described in online supplementary methods.

10.1136/gutjnl-2017-314190.supp1 Supplementary file 1

## Results

### Study population and colonoscopies

A total of 987 patients who underwent a total of 7516 endoscopies (number of colonoscopies=6985/7516 or 92.9%; see online supplementary figure S1) met the inclusion criteria. Of these, 679 procedures were performed prior to the index procedure (9.0%; see online supplementary figure S1). Including colonoscopies performed prior to the index procedure, the cumulative follow-up duration from the time of the first colonoscopy to the study end point was 13 884 patient-years. The remaining demographics of the study population is summarised in table 1.

**Table 1:** Demographics of study population (n=987)

Characteristic	Values
Male sex (n, %)	559 (56.6)
Age at UC diagnosis (median, IQR)	30 years (22–40)
Duration of UC at study end point (median, IQR)	29 years (21–38)
Extensive UC (n, %)	987 (100)
Primary sclerosing cholangitis (n, %)	42 (4.3)
Follow-up duration (median, IQR)	13 years (9–18)
Colonoscopy (median, IQR)	6 per patient (4–9)
Surveillance interval (median, IQR)	22 months (12–28)
Chromoendoscopy (n, %)	1056 (15.1%)
Biopsies (median, IQR)	9 (6–10)

**Figure 2:**
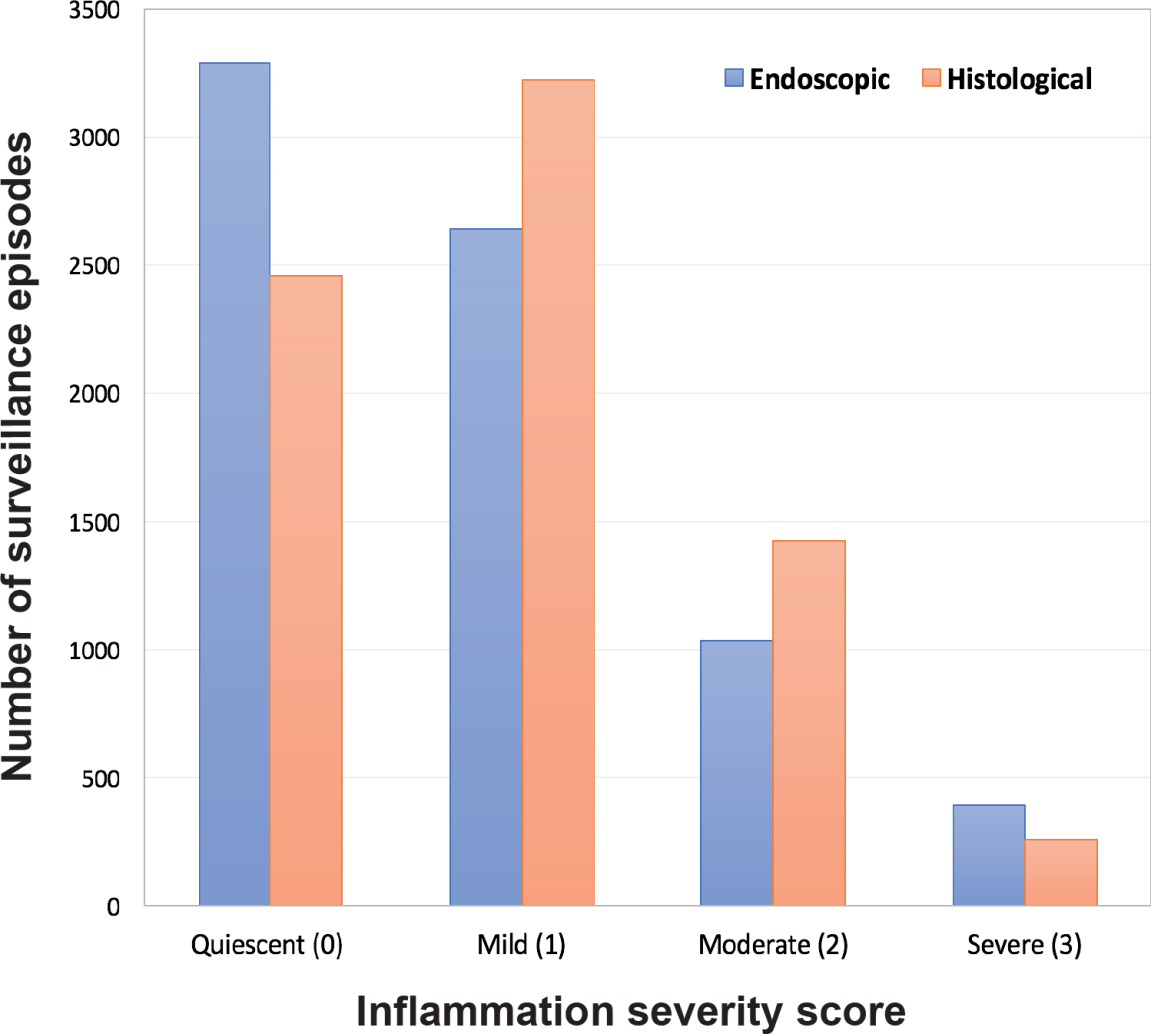
Endoscopic (blue) and histological (red) inflammation scores for each surveillance procedures. A significant proportion (66.6%) of surveillance episodes showed histological inflammatory activity (mild, moderate or severe).

A significant proportion (66.6%) of surveillance episodes showed histological inflammatory activity (mild, moderate or severe; figure 2). This was significantly higher compared with reported endoscopic inflammatory activity (55.4%; χ², P<0.001).

### Follow-up

Of the 987 patients, 783 patients (79.8% of study population) were still on active surveillance as of 1 July 2013. During the study period, 97 patients underwent colectomy. Indications for surgery were CRC in 14, dysplasia in 46 and medically refractory disease in 37 patients.

In addition, 22 patients (2.2% of study population) died, either due to CRC without surgery (1/22) or other unrelated diseases (21/22). The remaining 85 patients (8.6% of study population) left the surveillance programme prior to 1 July 2013 due to one of the following: age/comorbidities/defaulted (71/85), transfer of care to another hospital (11/85) and non-colorectal malignancy (3/85).

### Study end points

Overall, 97 patients (incidence rate, 7.9 per 1000 patient-years) developed at least one episode of CRN during the study period. The first episode of CRN was high-risk LGD in 65 (6.6 % of study population), HGD in 18 (1.8%) and CRC in 11 (1.1%). The first and maximal episode of CRN for these patients is shown in online supplementary figure S2.

### Factors determining development of colorectal neoplasia

#### Univariate analysis

The results of univariate analysis of potential demographic, endoscopic and histological risk factors associated with CRN development are shown in tables 2 and 3, respectively.

**Table 2:** Univariate analysis of potential demographic factors associated with colorectal neoplasia

Variables	Number (%)	HR (95% CI)	P value
Family history of colorectal cancer	48 (4.9)	0.5 (0.2 to 1.5)	0.2
Male sex	559 (56.6)	0.9 (0.6 to 1.4)	0.7
**Primary sclerosing cholangitis**	42 (4.3)	**3.5** (1.7 to 6.9)	**<0.001**
Duration of UC at colonoscopy (year: median, IQR)	19 (12–28)	1.02 (0.99 to 1.04)	0.2
**Age** (year: median, IQR)			
**At colonoscopy**	52 (42–61)	**1.03** (1.01 to 1.05)	**0.001**
At UC onset	30 (22–40)	1.02 (1.01 to 1.04)	0.01
5-aminosalicylate exposure (year)			
<1	183 (18.5)	1	0.6
1–10	448 (45.4)	1.2 (0.7 to 2.0)	0.5
>10	356 (36.1)	0.8 (0.5 to 1.4)	
Immunosuppressant exposure (year)			
<1	631 (63.9)	1	0.1
1–10	267 (27.1)	1.4 (0.9 to 2.3)	0.4
>10	89 (9.0)	1.3 (0.7 to 2.5)	

Statistically significant variables are highlighted in bold.

**Table 3:** Univariate analysis of potential histological and endoscopic factors associated with colorectal neoplasia

Variables	Number (%)	HR (95% CI)	P value
Cumulative inflammatory burden (median, IQR)			
Endoscopic*	10.2 (5.7–15.1)	**1.8** (1.4 to 2.4)	**<0.001**
Histological*	11.6 (7.2–16.0)	**2.0** (1.5 to 2.8)	**<0.001**
Macroscopic features of chronicity			
**Scarring only**	537 (54.4)	**2.6** (1.5 to 4.4)	**<0.001**
**Tubular, featureless or shortened colon**	318 (32.2)	**3.6** (2.2 to 5.9)	**<0.001**
**Postinflammatory polyps**	447 (45.3)	**2.6** (1.7 to 3.9)	**<0.001**
**Stricture**	46 (4.7)	**5.9** (2.4 to 14.5)	**<0.001**
**Procedure type**			
White-light (ref)	6543 (86.1)	1	
**Chromoendoscopy**	1056 (13.9)	**2.5** (1.5 to 4.1)	**<0.001**
Inadequate colonoscopy			
On preceding examination (n, %)	434 (5.9)	2.0 (1.0 to 3.9)	0.05
Mean (median, range)	0.0 (0.0–1.0)	5.2 (1.6 to 17.4)	0.01
**Mean number of biopsies** (median, IQR)	9 (7–10)	**1.2** (1.1 to 1.3)	**<0.001**
**Mean surveillance interval** (year; median, IQR)	2.1 (1.5–26.5)	**0.91** (0.89 to 0.94)	**<0.001**

*HR per **10-unit increase** in cumulative inflammatory burden (equivalent of 10, 5 or 3.3 years of continuous mild, moderate or severe active disease, respectively). Statistically significant variables are highlighted in bold.

**Figure 3:**
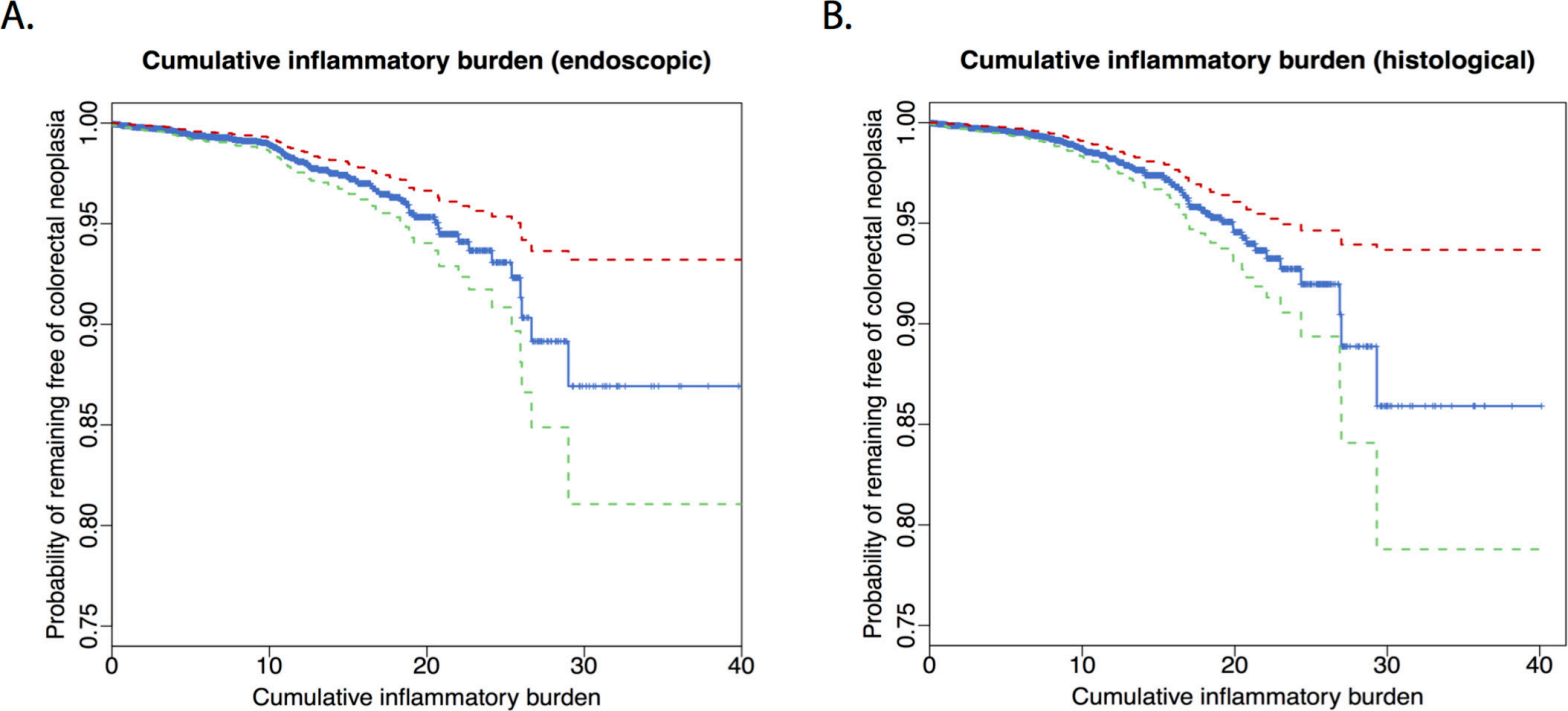
Kaplan-Meier plots showing the cumulative risk of colorectal neoplasia by endoscopic (A) or histological (B) CIB. Each 10 units of CIB are equivalent of 10, 5 or 3.3 years of continuous mild, moderate or severe active inflammation, respectively. Red and green dotted lines indicate 95% CI. CIB, cumulative inflammatory burden.

Both endoscopic (HR, 1.06 per 1-unit increase in CIB; 95% CI 1.03 to 1.09; P<0.001; figure 3A) and histological CIB (HR, 1.07 per 1-unit increase in CIB; 95% CI 1.04 to 1.11; P<0.001; figure 3B) were significantly associated with development of CRN (table 3). For every 10-unit increase in CIB, HR was 1.8 (95% CI 1.4 to 2.4; P<0.001) for endoscopic and 2.0 (95% CI 1.5 to 2.8; P<0.001) for histological CIB: as every 1-unit of CIB is equivalent of 1 year of mild active inflammation, these suggest that the risk of CRN approximately doubles for every 10 years of mild, 5 years of moderate or 3.3 years of severe active disease. In contrast, the duration of disease was not significantly associated with CRN development (HR, 1.02; 95% CI 0.99 to 1.04; P=0.2; table 2).

We observed a notable association between other endoscopic features of chronicity and the development of CRN, with a HR of 2.6 (95% CI 1.5 to 4.4; P<0.001) when scarring was present, rising to 3.6 (95% CI 2.2 to 5.9; P<0.001) when more prominent features, such as a tubular, featureless or shortened colon were found. In addition, postinflammatory polyps (HR, 2.6; 95% CI 1.7 to 3.9; P<0.001) and colonic strictures (HR, 5.9; 95% CI 2.4 to 14.5; P=0.001) were also significant factors for developing CRN (table 3).

#### Multivariate analysis

All variables that were significantly correlated with the development of CRN (at Bonferroni adjusted P<0.003) in univariate analyses were entered into the multivariate model. As endoscopic and histological CIB were significantly correlated to each other (Pearson’s correlation, 0.82, 95% CI 0.81 to 0.82; P<0.001), only one of these variables was entered into the multivariate model each time.

The final multivariate model is shown in table 4. After adjusting for patient’s age, average number of biopsies, surveillance interval and colonoscopy type (ie, white-light or chromoendoscopy; see online supplementary data for further discussions on these potential confounders), both endoscopic (HR, 1.08 per 1-unit increase in CIB; 95% CI 1.04 to 1.11; P<0.001) and histological CIB (HR, 1.08; 95% CI 1.03 to 1.12; P<0.001) were significantly associated with the development of CRN. Again, these indicate that the risk of CRN increases by 2.1-fold (95% CI 1.5 to 2.9; P<0.001 for endoscopic and 95% CI 1.4 to 3.0; P<0.001 for histological) for every 10 years of mild, 5 years of moderate or 3.3 years of severe active disease (table 4).

**Table 4:** Final multivariate model showing predictors associated with colorectal neoplasia

Variables	HR (95% CI)	P value
Cumulative inflammatory burden		
Endoscopic*	**2.1** (1.5 to 2.9)	**<0.001**
Histological*	**2.1** (1.4 to 3.0)	**<0.001**
Macroscopic features of chronicity		
Scarring only	1.4 (0.8 to 2.4)	0.3
**Tubular, featureless or shortened colon**	**1.8** (1.1 to 2.9)	**0.03**
Postinflammatory polyps	1.2 (0.8 to 1.8)	0.4
**Colonic stricture**	**3.2** (1.3 to 8.0)	**0.01**
Chromoendoscopy	1.0 (0.6 to 1.7)	1.0
**Primary sclerosing cholangitis**	**2.3** (1.1 to 4.7)	**0.02**
**Age (at colonoscopy)**	**1.02** (1.00 to 1.04)	**0.03**
**Average number of biopsies**	**1.14** (1.08 to 1.20)	**<0.001**
**Average surveillance interval**	**0.93** (0.91 to 0.96)	**<0.001**

*HR per 10-unit increase in cumulative inflammatory burden (equivalent of 10, 5 or 3.3 years of continuous mild, moderate or severe active disease, respectively). Statistically significant variables are highlighted in bold.

Colonic stricture (HR, 3.2; 95% CI 1.3 to 8.0; P=0.01) and other macroscopic features of disease chronicity (ie, tubular, featureless or shortened colonic appearance; HR, 1.8; 95% CI 1.1 to 2.9; P=0.03) and PSC (HR, 2.3; 95% CI 1.1 to 4.7; P=0.02) were also significant contributory factors for developing CRN (see online supplementary figure S3 A–C). On multivariate analysis, scarring (HR, 1.4; 95% CI 0.8 to 2.4; P=0.3) and postinflammatory polyps (HR, 1.2; 95% CI 0.8 to 1.8; P=0.4) were no longer significant.

### Developing pragmatic markers for CRN risk

The significant association between CIB and the risk of CRN highlighted an importance of considering *cumulative* effect of inflammation on colorectal carcinogenesis. Intuitively, this questions the validity of risk stratifying patients based only on the immediately preceding colonoscopy, as it only reflects severity of inflammation at a single time-point only. We sought to test this with our data—this revealed that neither endoscopic (HR, 1.0 per 1-unit increase in score; 95% CI 0.8 to 1.3, P=1.0) nor histological (HR, 0.9; 95% CI 0.7 to 1.2; P=0.5) severity of inflammation score based on immediately preceding colonoscopy were significantly associated with risk of CRN development (table 5).

**Table 5:** Efficacy of each markers of inflammation in predicting development of colorectal neoplasia after adjusting for other significant predictors

Inflammation severity (Median number of colonoscopies)	HR (95% CI)	P value
Endoscopic		
In last procedure only (1)	1.0 (0.8 to 1.3)	1.0
*Mean severity* in preceding		
**3 years** (2)	**1.4** (1.0 to 1.8)	**0.02**
**5 years** (3)	**1.5** (1.1 to 2.0)	**0.007**
**10 years** (4)	**1.7** (1.2 to 2.4)	**0.001**
**All episodes** (6)	**1.9** (1.3 to 2.6)	**2.3e-04**
*Max severity* in preceding		
3 years(2)	1.0 (0.8 to 1.2)	0.9
5 years(3)	1.1 (0.9 to 1.4)	0.4
**10 years** (4)	**1.3** (1.0 to 1.7)	**0.02**
**All episodes**(6)	**1.4** (1.1 to 1.8)	**0.003**
*Persistency* in preceding		
**3 years** (2)	**1.3** (1.1 to 1.6)	**0.01**
**5 years** (3)	**1.4** (1.1 to 1.8)	**0.001**
**10 years** (4)	**1.6** (1.2 to 2.0)	**4.1e-0.4**
**All episodes** (6)	**1.8** (1.4 to 2.4)	**1.1e-05**
Histological		
In last procedure only (1)	0.9 (0.7 to 1.2)	0.5
*Mean severity in preceding*		
**3 years** (2)	**1.5** (1.1 to 2.1)	**0.01**
**5 years** (3)	**2.2** (1.6 to 3.1)	**2.4e-06**
**10 years** (4)	**2.8** (1.9 to 4.1)	**5.8e-08**
**All episodes** (6)	**3.1** (2.1 to 4.5)	**4.8e-09**
*Max severity* in preceding		
3 years (2)	1.1 (0.8 to 2.4)	0.6
**5 years** (3)	**1.4** (1.1 to 1.8)	**0.01**
**10 years** (4)	**1.7** (1.3 to 2.2)	**7.0e-05**
**All episodes** (6)	**1.8** (1.4 to 2.4)	**4.7e-06**
*Persistency* in preceding		
**3 years** (2)	**1.5** (1.1 to 1.9)	**0.005**
**5 years** (3)	**1.9** (1.4 to 2.6)	**9.5e-06**
**10 years** (4)	**2.2** (1.6 to 3.0)	**7.3e-07**
**All episodes** (6)	**2.4** (1.7 to 3.3)	**1.2e–07**

All HRs presented are **per 1-unit increase** in each inflammatory score. For *mean* and *maximal severity*, each 1-unit increase represents increments from quiescent to mild (0–1), mild to moderate (1–2) or moderate to severe (2–3) active inflammation. For *persistenc*y, 1-unit increase represents 33.3% increase in proportion of surveillance episodes with active inflammation of any severity (ie, 0=0%, 1=33.3%, 2=66.6% and 3=100%). The median number of surveillance procedures performed in preceding 3-year, 5-year, 10-year period for each episode was 2 (IQR, 2–3), 3 (IQR, 2–4) and 4 (IQR, 2–5), respectively. Statistically significant variables are highlighted in bold.

These suggest that while risk stratification based on a single most recent procedure provides a rapid estimation of the CRN risk, its accuracy may not be adequate. On the other hand, risk stratification by CIB may not be practical in many clinical setting as it is time-consuming and often patient’s entire record of endoscopic surveillance may not be readily available.

To this end, we sought to investigate whether inflammation data obtained only from surveillance episodes, which occurred in the preceding 3, 5 or 10 years could be used to accurately estimate CRN risk. This could allow more rapid assessment of the risk as while appreciating the cumulative effect of inflammation by considering multiple surveillance procedures occurring over many years. Three inflammation scores were derived and are defined as below:

1. *Mean severity*: the sum of all severity scores obtained from all surveillance procedures performed within preceding *n* years (ie, 3, 5 or 10 years) divided by the total number of surveillance procedures performed within preceding *n* years (ie, 3, 5 or 10 years, respectively).
2. *Maximum severity*: the maximal inflammation score found in any of surveillance procedures performed within preceding *n* years (ie, 3, 5 or 10 years).
3. *Persistency*: the number of surveillance episodes with active inflammation (of any severity) divided by the total number of surveillance procedures performed within preceding *n* years (ie, 3, 5 or 10 years).

The statistical significance and predictive power for each of these markers after adjusting for other significant predictors aforementioned are illustrated in table 5. The median number of surveillance procedures performed in the preceding 3, 5 and 10 years for each surveillance episode was 2 (IQR, 2–3), 3 (IQR, 2–4) and 4 (IQR, 3–5), respectively.

There are four important points to note from table 5. First, the strength of association between inflammation severity scores (ie, mean and maximal inflammation severity) and CRN outcome became markedly stronger when inflammation data from more surveillance procedures occurring over longer preceding years were used to derive these scores (as evidenced by increasing HR and decreasing P values). This pattern was seen uniformly across all inflammation scores and indeed re-emphasises the importance of considering CIB in assessing the CRN risk.

Second, not only the inflammation severity but also the inflammation *persistency* were significantly associated with the risk of developing CRN. For every 33.3% increase in persistency of microscopic inflammation, the risk of CRN increased by 2.4-fold (95% CI 1.7 to 3.3; P=1.2e–07; table 5 & online supplementary figure S3D). Importantly, this association remained even when the model was adjusted for mean inflammation severity (HR, 1.7; 95% CI 1.1 to 1.7; P=0.02 for persistency and HR, 1.8; 95% CI 1.0 to 3.3; P=0.04 for mean severity).

Third, while many inflammation scores derived from endoscopic findings showed significant associations with CRN outcome, the strength of association was generally weaker compared with scores based on histology, as evidenced by uniformly lower HR and higher P value for equivalent scores.

Finally, the mean severity scores were consistently more closely associated with CRN outcome compared with equivalent maximal severity scores.

**Figure 4:**
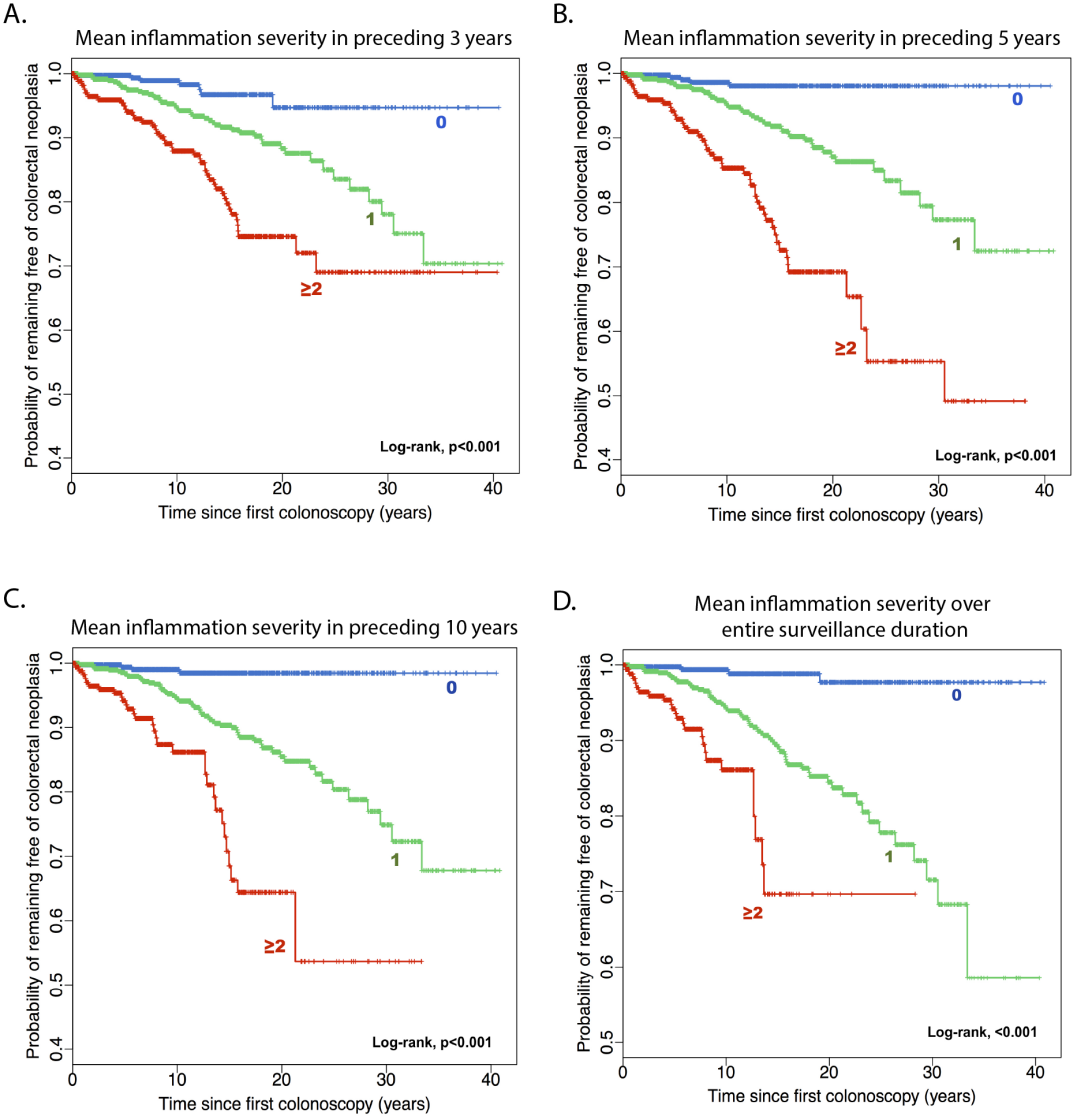
Kaplan-Meier plots showing the cumulative risk of colorectal neoplasia by mean severity of microscopic inflammation (rounded to nearest ones) in preceding 3 (A), 5 (B) and 10 years (C) or over an entire surveillance history (D).

Overall, the mean inflammation severity scores derived from histology showed the strongest association with the CRN outcome. The Kaplan-Meier plots showing the risk of CRN based on mean histological severity in the preceding 3, 5 and 10 years or over an entire surveillance history are shown in figure 4.

## Discussion

This is one of the largest detailed studies to date investigating individual risk factors for CRN in UC. We have demonstrated that the risk of CRN in UC is significantly associated with CIB. Our results show that an accurate estimate of CRN risk is unlikely to be achieved using only the most recent colonoscopy and requires assessment of multiple colonoscopies occurring over many preceding years to take the cumulative effect of inflammation into account.

### The risk of colorectal neoplasia is significantly associated with actuarial cumulative inflammatory burden

While the association between inflammation and carcinogenesis is clear, the model in which *how* inflammatory insults occurring over time contributes to the risk of CRN development is poorly understood. Our results strongly suggest that the risk of developing CRN is closely associated with the CIB, indicating that the neoplasia risk in these patients is positively correlated to the total amount of inflammatory damage accumulated over time.

From a clinical perspective, our findings have important implications on managing cancer risk in patients with IBD, as it questions the validity of (1) duration of colitis as a risk factor for neoplasia and (2) assessing the neoplasia risk based on single, most recent episode of colonoscopy.

### Duration of colitis is no longer a predictive marker for risk of colorectal neoplasia development

Studies suggested that the duration of colitis (or time since the colitis symptoms onset) is an important risk factor for developing CRC.^1 2 12^ It is important to note that these studies are old and were based on cohorts of patients who did not often have access to contemporary care that is available to a newer cohort of patients (better clinician and patient awareness and availability of biological agents, for example), resulting in a higher proportion of patients having persistently active disease compared with contemporary patients. Thus, it is possible that the disease duration was in fact, a *proxy* measure for the CIB that is, as seen in our study, significantly associated with risk of developing CRN.

This is unlikely to be true with the current generation of patients who were exposed to contemporary care, ultimately resulting in a higher proportion of patients with well-controlled disease as many of them would have prolonged periods of quiescent disease (therefore low CIB). As a result, the duration of colitis may no longer serve as a proxy measure for CIB. Indeed, the disease duration failed to show any significant association with development of CRN in our data.

### Risk stratification should involve assessment of multiple surveillance procedures occurred over several years in patient’s surveillance history

The current British Society of Gastroenterology guideline recommends risk stratification based on inflammatory activity present at the most recent surveillance episode.^8^ This strategy may overlook any significant inflammatory insults that had occurred previously and thus may not accurately depict an individual patient’s history of inflammatory exposure. In support of this, severity of inflammation on the immediately preceding procedure alone failed to demonstrate any significant association with CRN (table 5).

The importance of considering CIB was further highlighted by the fact that the predictive accuracy of inflammation severity markers (ie, mean severity, max severity and persistency) improved in a linear fashion by including more surveillance procedures in calculating these scores. Thus, our data suggest that an adequate neoplasia risk assessment for patients undergoing surveillance must involve assessment of multiple surveillance procedures occurring over many previous years.

### Developing a pragmatic risk stratification strategy

Although it is clear that the most accurate risk estimation may only be achieved by assessing a patient’s entire surveillance history, this is often impractical in busy clinical practice. So how do we meet a balance between practicality and efficacy when using data on inflammation for risk stratification? The results from our study suggest a potential strategy.

First, the severity of inflammation score for each colonoscopy episode may be derived *only* from the segment with worst disease, as we did in our study, instead of assessing the whole colon and calculating its average severity (a method used in previous studies^4–6^). Although this may result in overestimation of overall colonic inflammation (eg, patient with moderately active disease only in a single segment with quiescent disease elsewhere would still be given an overall score of 2), we believe that an overestimation of CRN risk resulting in increased false positive rate is likely to be safer than having more false negatives from underestimating the CRN risk.

Second, the *mean* severity score calculated using *histology* reports from the surveillance procedures performed in preceding 5 or 10 years may offer a reasonable balance between the accuracy and practicality. This was based a number of observed trends in our study.

1. Mean severity score was easier to calculate than CIB score.
2. Mean severity score based on histology was more accurate compared with the score based on endoscopy.
3. Mean severity scores were significantly more accurate than maximum severity scores or persistency.
4. Calculating score based on preceding 5 or 10 years worth of surveillance episodes only is significantly less burdensome compared with that of assessing entire surveillance records, although at a modest compromise in accuracy (table 5).

### Patients with persistently active colitis are at high risk of developing colorectal neoplasia regardless of inflammation severity

Previous studies demonstrated that markers of previous inflammatory activity such as postinflammatory polyps were significantly associated with risk of CRN.^13 14^ These data suggest that chronic inflammatory activity may play an important role for carcinogenesis in addition to the acute severe episode of inflammatory activity.

Indeed, in addition to the severity of inflammation, the persistency of inflammation was significantly associated with the risk of developing CRN in our study. Furthermore, this association remained significant even after adjusting for the mean severity. This result strongly suggests that patients with more persistent, chronically active colitis are at risk of developing CRN irrespective of inflammation severity.

Thus, cancer risk management should involve: (1) early identification of patients with persistently active disease, perhaps aided by other clinical and biochemical markers of chronic activity, such as clinical activity index (CAI)^15^ and calprotectin,^16^ respectively and (2) rigorous treatment of active disease to control symptoms and to achieve complete histological mucosal healing.

In our study population, histological activity was seen in a substantial proportion (66.6%) of surveillance procedures and a significant proportion of patients (27.7%) suffered constantly active disease (ie, with 100% persistency), indicating that there is a considerable room for further improvement in cancer risk management by optimising their medical therapy.

### Macroscopic appearance: features of chronicity, postinflammatory polyps and stricture

It is recognised that there is a wide spectrum of chronic endoscopic changes in patients with UC, such as scarring, postinflammatory polyps and a tubular, featureless colon. However, data on their significance with regard to the CRN risk are scarce, except for colonic strictures.^17–19^ Our updated analysis shows that a colonic stricture and a tubular, featureless or shortened colonic appearance—which signifies persistent, chronically active disease—were independent risk factors for developing CRN. These findings again highlight the importance of considering persistency of inflammation when assessing CRN risk.

### Limitations

This study has important limitations. The most important and major limitation is the fact that this was a retrospective study and the data used in this study relied on accurate documentation at the time of the surveillance procedures. In particular, the endoscopic and histological inflammation scores were based on an overall impression of the reporting endoscopist/pathologist at the time of examination and were not separately validated. Thus, the possible effects of interobserver variability in grading severity of inflammation have not been fully taken into account. There is an important need for a validation study with similar design but with validated endoscopic and histological inflammation grading.

Second, while we tried to capture the changing nature of inflammation severity over time using time-dependent model, this nevertheless relied on an assumption that the degree of inflammation seen at colonoscopy was representative of the patient’s history of inflammation exposure. That is, any change in severity of inflammation that occurred during the intervening period between examinations would not have been captured.

This is of particular issue for CIB, since it inherently assumes that the degree of inflammation remains constant during each surveillance interval, which may not be necessarily the case in real life. To have more complete roadmap of patient’s inflammatory activity within the interval, one should consider using other clinical indices such as the clinical Mayo score^20^ or CAI^15^ as well as biochemical indices including calprotectin^16^ and lactoferrin.^21^ Therefore, these are important revenues for future research.

## Conclusion

In summary, we showed that the CIB is strongly associated with the risk of developing CRN in patients with UC. Reflecting this, the severity of inflammation at the last colonoscopy failed to show significant association with CRN risk. This indicates that accurate risk stratification requires assessment of multiple surveillance procedures performed in many preceding years to take a patient’s CIB into account. The mean severity score (based on the segment worst affected by colitis only) averaged over the preceding 5 or 10 years may offer a rapid calculation of the risk for use in routine clinical practice. Finally, patients with severe and persistent inflammation should be medically optimised with the aim of achieving mucosal healing and prioritised to undergo more intensive surveillance protocols with advanced endoscopic techniques to allow the timely detection and early intervention for CRN.
