# Supplementary material for: Cumulative burden of inflammation predicts colorectal neoplasia risk in ulcerative colitis: a large single-centre study
Source: Gut. 2017 Nov 17;68(3):414–22. doi: 10.1136/gutjnl-2017-314190 (PMC6581019; doi:10.1136/gutjnl-2017-314190)
Supplement: Supplementary file 1 [file gutjnl-2017-314190supp001.docx]

**supplementary data**

**Cumulative inflammation predicts risk of colorectal neoplasia in ulcerative colitis**

This supplementary file includes some of the important details on methodology and results that were removed from the original manuscript due to word count restrictions.

Table of Contents

[1. Supplementary methods 3](#_Toc497596988)

[***Construction of multivariate model and handling of variables in time-dependent Cox-regression model*** 3](#_Toc497596989)

[2. Notes on age, mean number of biopsies taken, surveillance interval and chromoendoscopy 5](#_Toc497596990)

[**Supplementary results** 5](#_Toc497596991)

[**Discussion: age and duration of colitis** 6](#_Toc497596992)

[**Discussion: number of biopsies and surveillance interval** 7](#_Toc497596993)

[3. Supplementary Figures 8](#_Toc497596994)

[4. References 12](#_Toc497596995)

# **1. Supplementary methods**

## ***Construction of multivariate model and handling of variables in time-dependent Cox-regression model***

The association between each variable and the study end points was examined using time-dependent Cox proportional hazards methods with right-censored data. As 18 predictors were being tested against two outcomes, a *Bonferroni* adjusted significance level (p<0.003) was used to select variables for entering into the multivariate model to correct for multiple testing. The Akaike information criterion (AIC) was then used to identify the model that provided the best fit while being the most parsimonious^1^.

The following variables were considered to remain constant over time, and thus coded as static variables: age of ulcerative colitis (UC) onset, sex, concomitant primary sclerosing cholangitis (PSC), and family history of colorectal cancer (CRC). In addition, exposure to 5-aminosalicylates (5-ASAs) and immunosuppressants were coded as static variables. This was because the only high quality information available was the duration of drug exposure by the end of follow-up. Thus, their primary purpose was to control for their effect on other predictors if required, rather than investigating them as a potential risk/protective factor for progression to colorectal neoplasia.

All other variables were coded as time-dependent covariates. In other words, the values of these time-dependent covariates for each patient were updated at each episode of surveillance, which ensures that the regression model only considers the values up to and including the time of particular comparison. Therefore, each patient may be placed on a different risk category depending on where they are in their follow-up. The detailed information on how each of these variables is categorised and handled in a time-dependent Cox-model is described below.

1. ***All inflammation scores*** (i.e. cumulative inflammatory burden, mean severity, maximum severity and persistency) were time-dependent covariates and thus updated at each surveillance episodes as described above.
2. ***Post-inflammatory polyps and colonic stricture***: their presence or absence was recorded for each episode of colonoscopy. Once reported, they were assumed to be present in all subsequent colonoscopies.
3. ***Macroscopic features of chronicity***: this was a categorical variable which was defined as follows – 1) no features of chronicity, 2) scarring only, and 3) tubular, featureless, or shortened colonic appearance. These were again assumed to be present in all subsequent colonoscopies once reported.
4. ***Inadequate colonoscopy***: each episode was considered to be inadequate if cecal intubation had not been achieved or mucosal views were inadequate due to poor bowel preparation (flexible sigmoidoscopy episodes were not considered). We assessed the criteria against CRN outcome: a) the adequacy of the immediately preceding colonoscopy (binary variable – i.e. adequate or inadequate) and b) the proportion (%) of colonoscopies considered inadequate up to the date of comparison (continuous variable).
5. ***Mean number of biopsies*** (continuous variable)*:* sum of all biopsies taken during each patient’s surveillance history divided by the number of surveillance procedures patients underwent within that time.
6. ***Mean*** ***surveillance interval*** (continuous variable): for each patient, this value corresponds to number of years the patient had been under surveillance divided by number of surveillance procedures patient underwent during that time.

# **2. Notes on age, mean number of biopsies taken, surveillance interval and chromoendoscopy**

These variables are important confounders to consider and indeed showed strong association with development of CRN in our study. Supplementary results and possible explanations regarding these findings are discussed below.

## **Supplementary results**

In the univariate analysis, the age at the time of colonoscopy (HR, 1.03 (per 1 year increase); 95% CI, 1.01-1.05, p=0.001), but not the age of UC symptom onset (HR, 1.02; 95% CI, 1.01-1.04, p=0.01), was significantly associated with CRN, after correcting for multiple testing (at *Bon-ferroni* adjusted p). The increasing average number of biopsies taken at colonoscopy (HR, 1.2 (per 1 biopsy increase); 95% CI, 1.1-1.3; p<0.001) and the use of chromoendoscopy (HR, 2.5; 95% CI, 1.5-4.1; p<0.001) was positively associated with CRN detection (main manuscript, Table 3). Conversely, increasing average surveillance interval (i.e. average number of years between surveillance procedures) demonstrated a negative association with the CRN detection (HR, 0.91 (per 1 year increase); 95% CI, 0.89-0.94; p<0.001; main manuscript, Table 3).

In multivariate analysis, only age at the time of colonoscopy (HR, 1.02; 95% CI, 1.00-1.04; p=0.03), average number of biopsies (HR, 1.14; 95% CI, 1.08-1.20; p<0.001) and average surveillance interval (HR, 0.93; 95% CI, 0.91-0.96; p<0.001) remained significant (main manuscript, Table 4).

## **Discussion: age and duration of colitis**

Similarly to the CRC occurring in colitis-free population^2^, patient’s age (at the time of colonoscopy) showed independent association with CRN development. In contrast, although the age of colitis onset had previously been reported as a risk factor for CRC^3–5^, it failed to show significant correlation with CRN development in our study after correcting for multiple testing. Furthermore, in agreement with our recent report^6^ and recent Danish cohort study^7^, increased duration of colitis was not an independent risk factor for development of CRN. Thus, our data do not support a policy of performing more frequent surveillance on the basis of increased disease duration per se – although they should be monitored with an appropriate interval on basis of older age.

## **Discussion: number of biopsies and surveillance interval**

Even after multivariate analysis, the number of biopsies taken during colonoscopy remained positively correlated with CRN being detected in subsequent surveillance procedures (this variable was measured as time-dependent covariate). Similarly, increasing surveillance interval was negatively correlated with CRN being detected in subsequent procedures.

Although these data appear to suggest, for example, benefit of taking more biopsies in detecting subsequent CRN, more likely explanation for these finding is that a form of detection bias had occurred. Patients who are judged to be at high-risk (e.g. those with previous dysplasia who have higher risk of developing further dysplasia) are likely to have more intensive surveillance with shorter surveillance interval and higher number of biopsies taken at each procedure. Thus, such protocol is preferentially select patients who already have a high chance of developing CRN, making them appear as if they have significant effect on CRN outcome. Nevertheless, even after adjusting for these confounders, inflammation scores, macroscopic appearance (tubular, featureless or shortened colon), colonic stricture and PSC were still significant contributory factors for CRN development (main manuscript, Table 4).

# **3. Supplementary Figures**


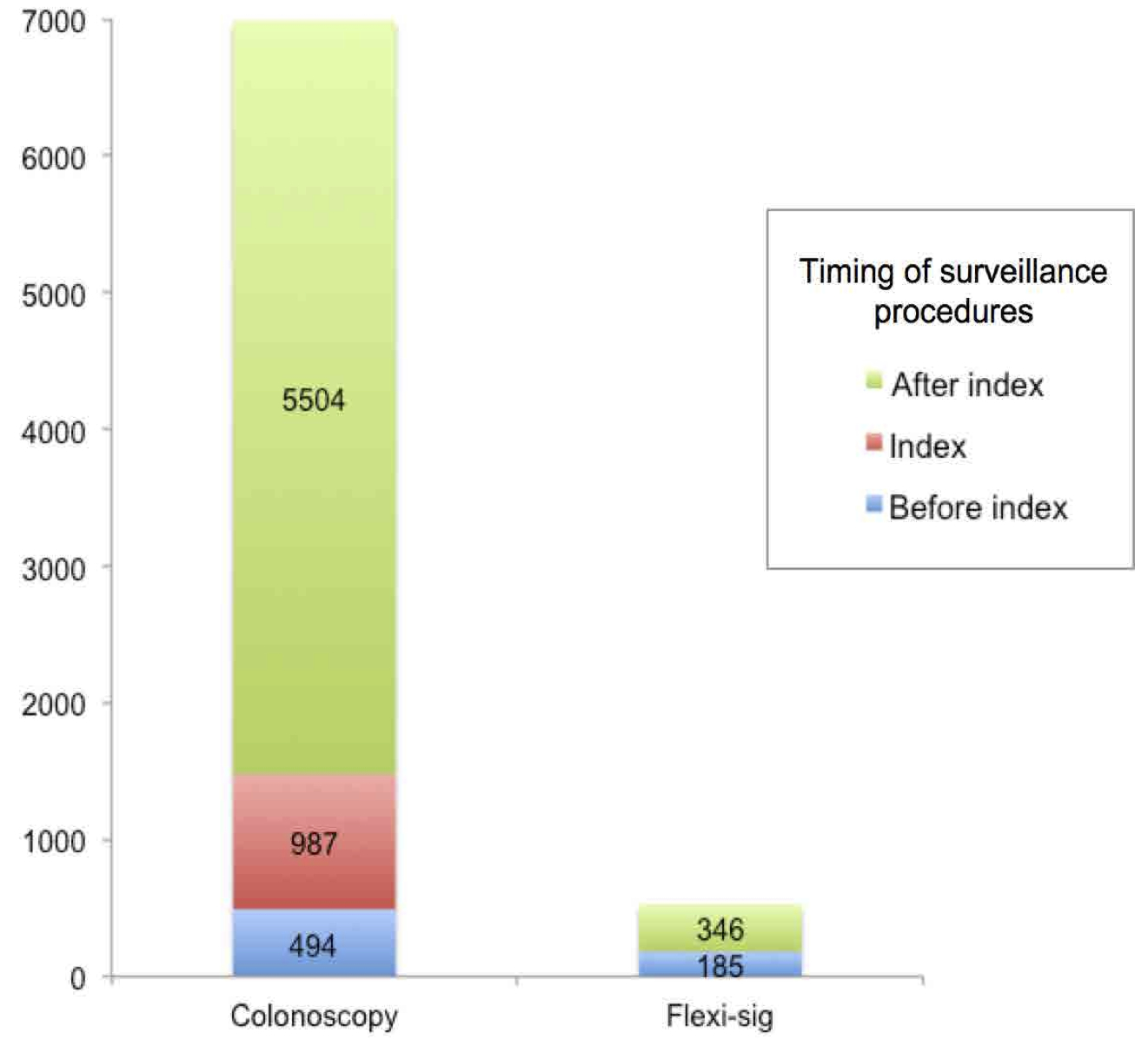


**Figure S1**: Type and timings of surveillance procedures in relation to the index colonoscopy (the first colonoscopy performed at our center after the patient entering the surveillance program). Flexi-sig = flexible sigmoidoscopy.


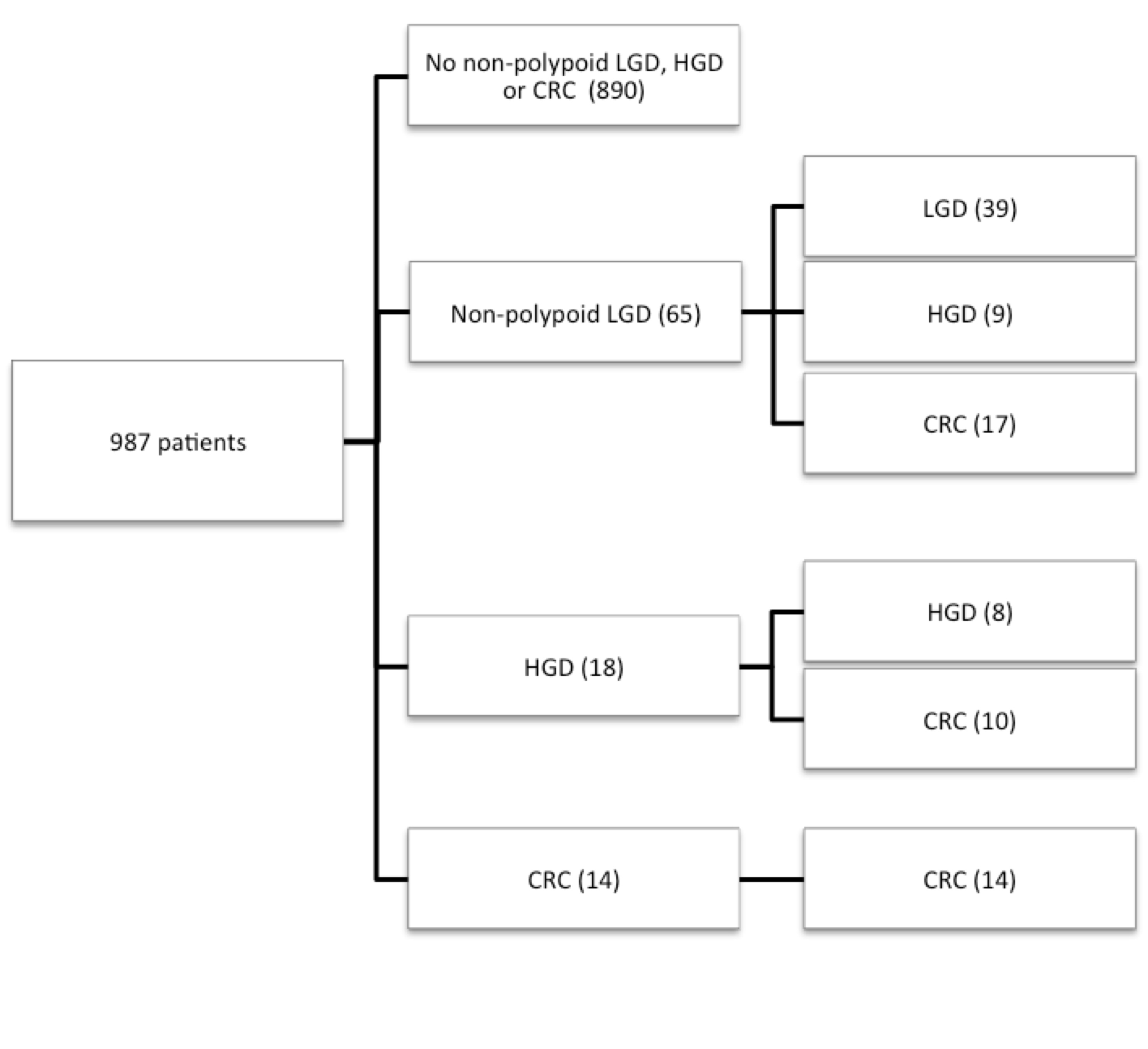


**Figure S2**: Colorectal neoplasia outcomes for the study cohort. Sixty-five patients (6.6% of study population) initially developed high-risk LGD (i.e. non-polypoid or large). Of these, 26 patients (26/64 or 40.0%) developed HGD (n=9) or CRC (n=17). In addition, a further 20 (20/64 or 31.3%) developed multifocal non-polypoid LGDs. Thus, overall, 46 of 64 (71.9%) patients who initially developed high-risk LGD developed either multifocal (non-polypoid) dysplasia, HGD or CRC. Eighteen patients (1.8% of study population) developed HGD without a preceding high-risk LGD episode. Of these, 2 had preceding polypoid LGD, and another 3 had a prior history of indefinite dysplasia. The remaining 13 patients had no neoplasia detected previous to HGD diagnosis. Eleven patients (1.1% of study population) developed CRC during their surveillance without any previously detected dysplasia. In addition, 3 patients developed CRC at a median of 4 years (range, 2-5) after leaving the surveillance program; these patients underwent a median of 6 colonoscopies (range, 4-13) prior to leaving the program in our centre.

**Figure S3**: Kaplan-Meier plots showing the cumulative risk of colorectal neoplasia by colonic stricture (A), macroscopic features of chronicity (B), primary sclerosing cholangitis (C) and microscopic inflammation persistency (D). All of the below variables were significantly associated with risk of developing colorectal neoplasia at multivariate analysis. HR = hazard ratio; 95% confidence intervals are shown in brackets.

**(A**)


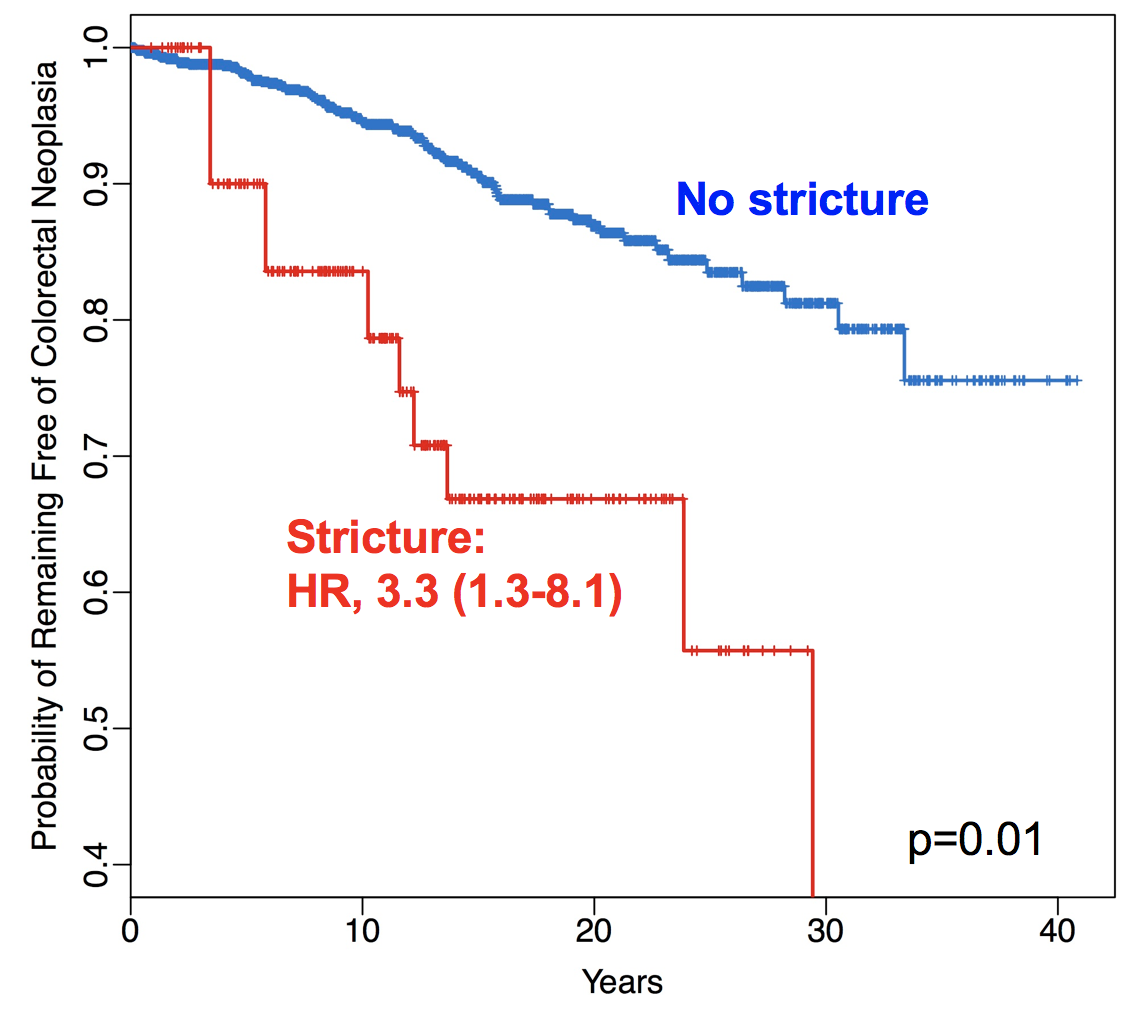


**(B)**


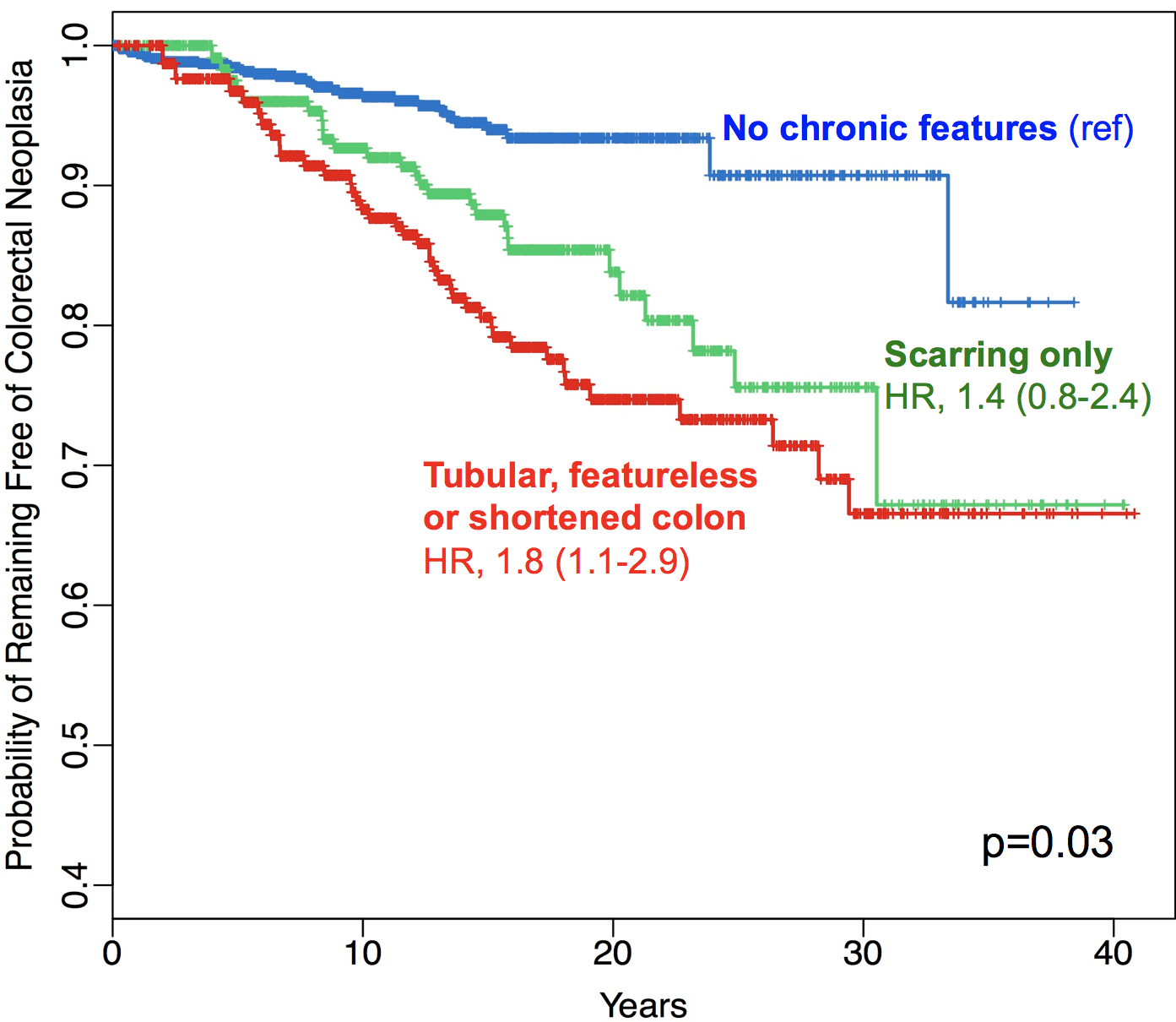


**(C)**


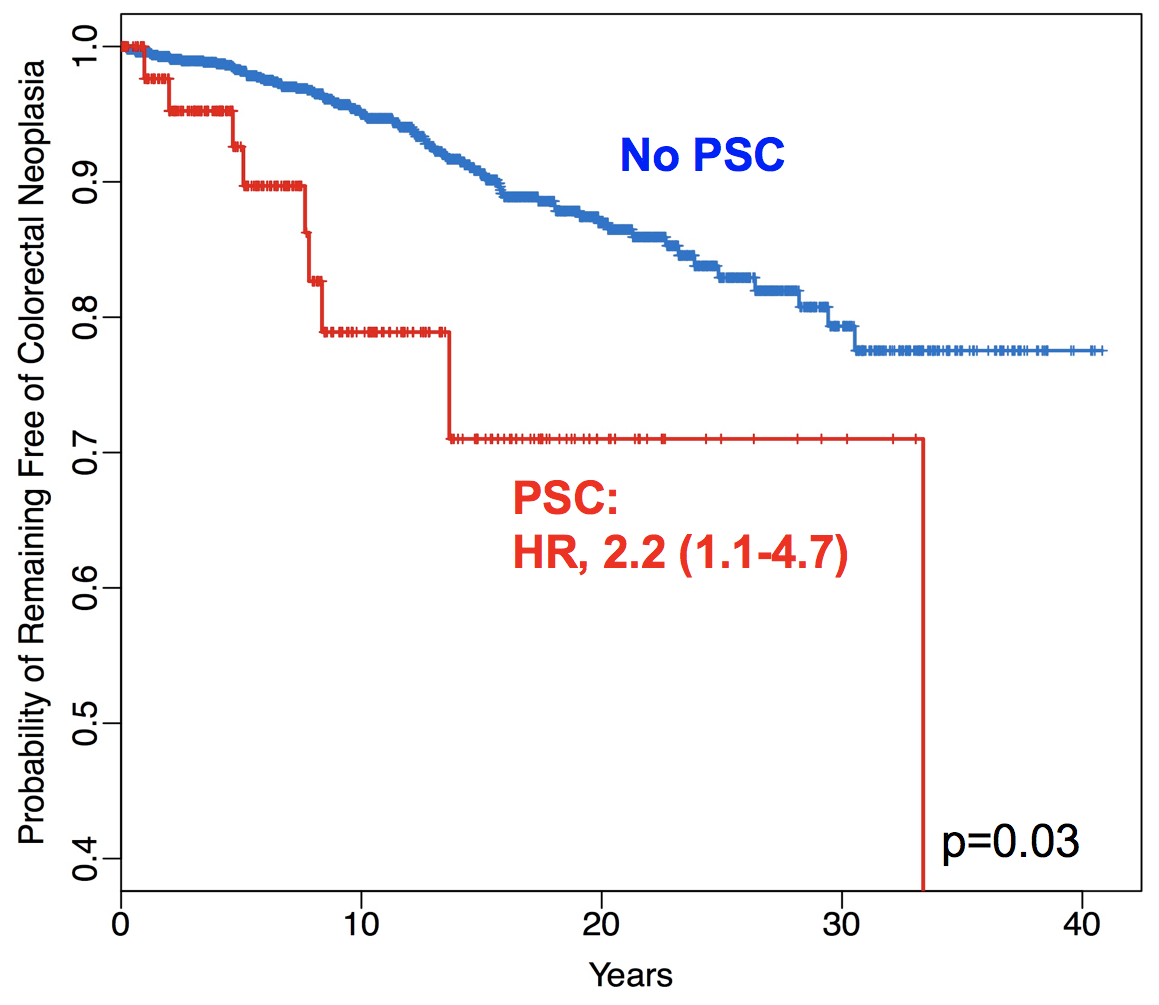


**(D)**


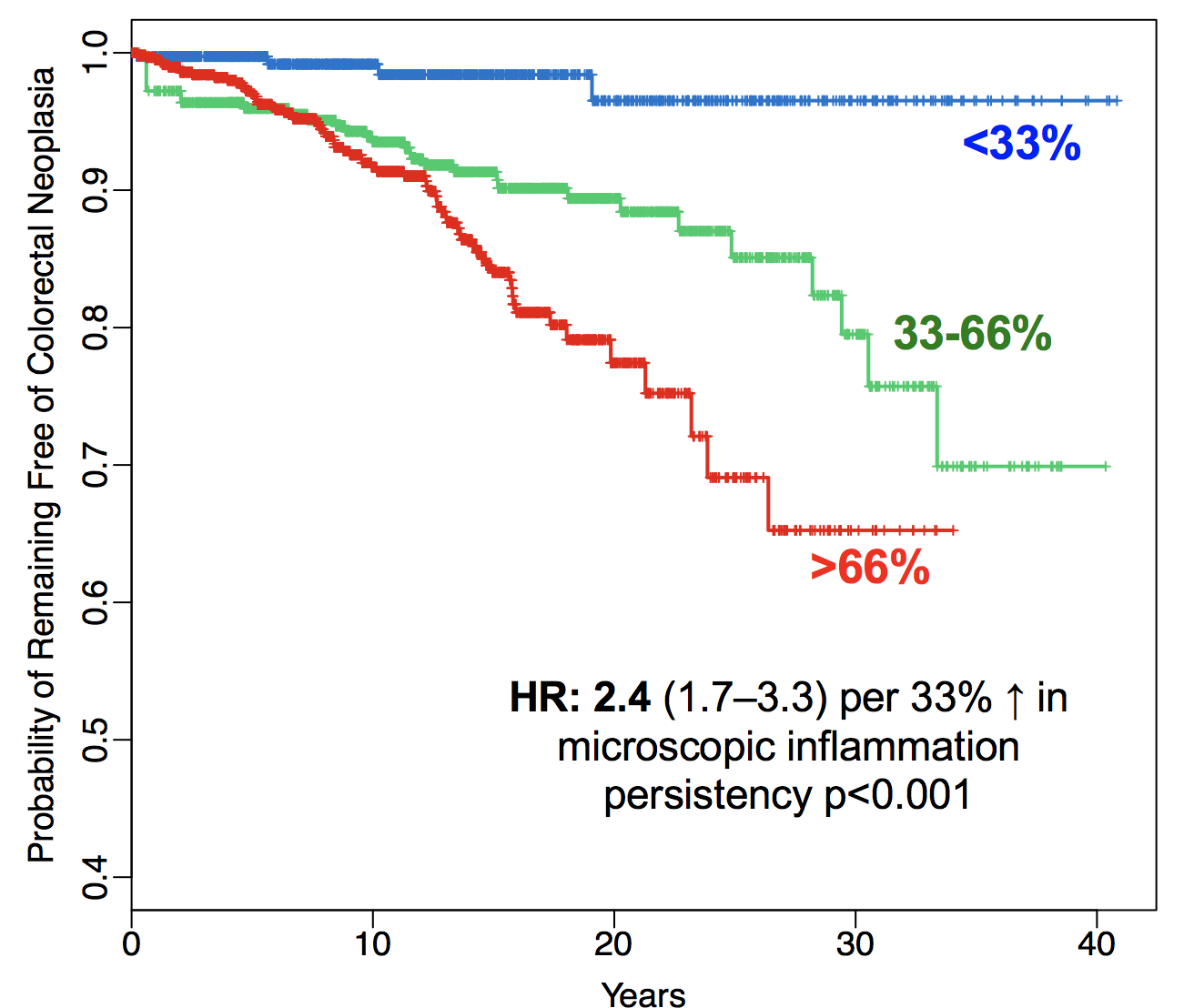


# **4. References**

1. Akaike H. Information Theory and an Extension of the Maximum Likelihood Principle. In: ; 1998:199–213.

2. Howlader N, Noone AM, Krapcho M, Garshell J, Miller D, Altekruse SF, Kosary CL, Yu M, Ruhl J, Tatalovich Z, Mariotto A, Lewis DR, Chen HS, Feuer EJ CK (eds). SEER Cancer Statistics Review, 1975-2011. Natl. Cancer Inst. 2013.

3. Devroede GJ, Taylor WF, Sauer WG, et al. Cancer risk and life expectancy of children with ulcerative colitis. N. Engl. J. Med. 1971;285:17–21.

4. Ekbom A, Helmick C, Zack M, et al. Ulcerative colitis and colorectal cancer. A population-based study. N. Engl. J. Med. 1990;323:1228–33.

5. Sugita A, Sachar DB, Bodian C, et al. Colorectal cancer in ulcerative colitis. Influence of anatomical extent and age at onset on colitis-cancer interval. Gut 1991;32:167–9.

6. Choi C-HR, Rutter MD, Askari A, et al. Forty-Year Analysis of Colonoscopic Surveillance Program for Neoplasia in Ulcerative Colitis: An Updated Overview. Am. J. Gastroenterol. 2015;110:1022–1034.

7. Jess T, Simonsen J, Jørgensen KT, et al. Decreasing risk of colorectal cancer in patients with inflammatory bowel disease over 30 years. Gastroenterology 2012;143:375-81–4.
